# Testicular self examination among Bahir Dar University students: application of integrated behavioral model

**DOI:** 10.1186/s12885-017-3935-8

**Published:** 2018-01-04

**Authors:** Hordofa Gutema, Yamrot Debela, Bizuayehu Walle, Kidist Reba, Habtamu Wondiye

**Affiliations:** 1 Department of Health Promotion and Behavioral Sciences, School of Public Health, College of Medicine and Health Science, Bahir Dar University, Bahir Dar, Ethiopia; 2 Department of Physiology, School of Medicine, College of Medicine and Health Science, Bahir Dar University, Bahir Dar, Ethiopia; 3 Department of Adult Health Nursing, School of Health Sciences, College of Medicine and Health Science, Bahir Dar University, Bahir Dar, Ethiopia

**Keywords:** Testicular self-examination, Integrated behavioral model, Path analysis, University students

## Abstract

**Background:**

Though the incidence of Testicular cancer among young is rising, little attention is given to promoting testicular self-examination which is recommended for its early prevention in developing countries**.** This study aimed to assess testicular self-examination and associated factors among Bahir Dar University students using integrated behavioral model.

**Methods:**

Cross sectional study was conducted among Bahir Dar University students in September, 2016. Systematic sampling technique was used to select 884 participants. Data was collected using self-administer questionnaire, entered into EPI Data 3.1 and exported to SPSS 21 for analysis. Path analysis was done using STATA 14.2 to check causal effect of integrated behavioral model constructs on testicular self-examination. Internal reliability of the items was checked using Cronbach’s alpha. Multivariable linear and Logistic regression were used to predict the role of independent variable on Intention and TSE respectively. Findings with *p*-value <0.05 at 95% confidence interval were considered as statistically significant in the final model.

**Results:**

Only 11.8% of the students practiced testicular self-examination in the previous year. Experiential and Instrumental attitude, Perceived control and Self-efficacy were significantly predicted behavioral intention with β coefficient 0.33, 0.12, −0.08 and 0.36. Students’ academic unit [OR = 0.31, 95% CI: 0.15–0.63], educational status of student’s father **AOR = 2.25; CI: 1.15–4.44]** and **[AOR = 3.00; CI: 1.36–6.64],** Intention [OR = 1.2, 95% CI: 1.1–1.31], Know-how of TSE [OR = 3.35, 95% CI:1.94–5.80] and knowledge **[AOR = 3.93; CI: 2.30–6.72]** were the significant predictors of testicular self-examination. The finding of path analysis also demonstrated as Experiential and Instrumental attitude, Perceived control and Self-efficacy have significant effect on intention with path coefficient of 0.33, 0.12, −0.07 and 0.36. Intention, Knowledge and Know-how have effect on testicular self-examination with path coefficient of 0.2, 0.36 and 0.22 respectively.

**Conclusions:**

Magnitude of testicular self-examination is low among university students and it is a product of the type of the college, family educational status, intention, Know-how and knowledge. So, behavior change communication strategy that focus on these behavioral factors should be designed and implemented to improve students’ regular practice of testicular self-examination.

**Electronic supplementary material:**

The online version of this article (10.1186/s12885-017-3935-8) contains supplementary material, which is available to authorized users.

## Background

Cancer of the testicle is the most frequently occurring cancer among younger men between age of 20–45 though it is uncommon type of cancer [1]. Testicular cancer (TC) begins when normal cells in a testicle change and grow uncontrollably and form a tumor. Germ cell tumor which develops in the sperm producing cell is the most common type of testicular cancer by accounting around 95%. Painless lump swelling in the testicles, a dull ache in scrotum and feeling of heaviness in the scrotum are the most common symptom of TC. Having family history of testicular cancer and being born with undescended testicles are expected to increase the risk of developing TC among men [2].

Even though evidence indicated raising of TC incidence in developed countries; especially among white in the last 5 decades and its low incidence in Asian and African countries made the scholars conclude geographic difference and being white as reason for increase of its incidence, the most recent finding shows raising of TC incidence among black American. So, there is no ground for non-increase of its incidence in African countries but lack of evidence [3–5]. Additionally, although the occurrence and death due to TC is estimated to be rare as compared with other cancers, recent research finding shows the chance of developing all prostate cancer in the late age is higher among men who ever experienced TC [6, 7] .

Furthermore, losing a single life of younger men who are in productive age group due to TC; which can be easily controlled has big impact on the family, the community and one’s country. It will also be additional burden of existing communicable and non-communicable disease in sub Saharan Africa like Ethiopia. So, intervention program which is based on evidence is very crucial to prevent and control this health problem.

According to World Health Organization, the burden of any type of cancer can be controlled and reduced through implementation of evidence based strategies for prevention, early detection and management of the patient [8]. Testicular cancer is highly curable if it detected early and appropriate treatment is given even after it is disseminated. The 5-year survival rate becomes 99.2% if the cancer has not spread outside the testicle while if it has spread into nearby lymph nodes, the rate become 96.1% and if it has spread to organs or lymph nodes away from the testicle, the five-year survival rate will be 73.2% [7]. Testicular self-examination (TSE) is a form of early diagnosis of TC which is recommended to be done by men’s above 15 years of age once a month after warm bath. But different research evidence shows very small number of men are practicing it and have intention to practice it [8–11].

Indeed, in Ethiopia, there is no clear evidence on TC including its incidence. However, some finding of study conducted in Africa reported TC incidence rate as less than 1% per 100,000 man-years [4, 5]. Despite its appropriateness and applicability in low resource setting and place where there is no effective screening method and treatment for TC, there is no apparent intervention program focusing on promoting TSE practice among younger men in Ethiopia. So, since it’s incidence is raising worldwide, this is appropriate time when the country should implement evidence based intervention program on TC.

Integrated Behavioral Model (IBM) is the behavioral model which developed in 1990s for further extension of Theory of Planned Behavior (TPB). For both of these models, the most important determinant of behavior is intention perform the behavior; however, IBM includes 3 other constructs that are not utilized within the TPB. According to IBM, in addition to intention, a particular behavior is most likely to occur if [1] a person has knowledge about, 2) there is no environmental constraint preventing performance, [3] the person has performed the behavior previously. The model also asserts, Direct determinants of individuals’ behavioral intention (BI) are their Instrumental and experiential attitudes, injunctive and descriptive norms, self-efficacy, and perceived control [12, 13]. Even though this model considered additional constructs to measure behavior, different scholars have preferred using TPA as conceptual framework to predict Behavioral Intention (BI) and TSE [10, 14, 15].

Since University students are on appropriate age for initiation of TSE, knowing the level of their BI and TSE practice is crucial for implementation of intervention. Hence, this study is aimed to determine TSE and associated factors among Bahir Dar University students using IBM as a conceptual framework.

## Methods

### Study design and population

A cross sectional study designed was conducted among male Bahir Dar University students. Bahir Dar University is found in Bahir Dar city which is located on northwest and 565 Kilo Meters away from Addis Ababa; the capital of Ethiopia. It is now among the largest Universities in the Federal Democratic Republic of Ethiopia, accepting more than 35,000 students in its 57 undergraduate and 39 graduate programs. Currently it has four campuses with four Colleges, three Institutes, three Faculties and one School.

### Participants and sampling

After excluding students who were enrolled in postgraduate, distance and extension program, all selected students who were present during the period were participated in the study. Sample size was calculated using single population proportion formula by assuming 95% confidence interval, 5% margin of error and 50% proportion of TSE. Adding 10% possible non-response rate and 2 design effect for sampling procedure, the sample size was determined to be 884 participants.

A multi-stage sampling procedure was employed to select study participants. Three academic unit from the eleven were selected randomly using lottery method as primary sampling unit. After allocating the calculated sample size proportionally to size of each selected academic unit, one department were selected from each. Then, simple random sampling was applied to select participants using sampling frame developed for selected departments as secondary sampling unit.

### Data collection procedure

The study was conducted from September 10 to 30, 2016 using self-administered structured questionnaire which was adapted from other studies on TSE [10, 15]. In addition, the finding of elicitation study which is critical step of applying IBM to identify relevant behavioral outcomes, referents, and environmental facilitators and barriers for practicing behavior using open-ended interview guide was used to develop the items [13]. Six college graduate data collectors and two supervisors who are health professional were recruited. Two-day training was given for data collectors and supervisors on the quality of the data and the procedure they have to follow during the data collection.

### Instruments

The instrument was comprised of Socio demographic characteristics, Knowledge (14 items), TSE (2 items), Intention (4 items of five point Likert scale), Direct measure of experiential attitude (EA) (4 semantic differential seven-point scale), indirect measure of EA (6 items of five point Likert scale), Direct measure of instrumental attitude (IA) using 5 semantic differential seven-point scale, Indirect measure of IA (8 items of five point Likert scale), Injunctive norm (IN) using 5 Items of five point Likert scale, Descriptive norm (DN) using 5 Items of five point Likert scale, Perceived control (PC) using 5 items of likely – unlikely scale, Self-efficacy (SE)10 items of five point Likert scale and environmental constraint (EC) (4 items) (Additional file 1). Negatively worded items were reversely coded before attempt of any analysis. Internal consistency of each construct’s items was checked using Cronbach’s alpha (α).

### Statistical analysis

The data was entered into Epi data 3.1 and analyzed using SPSS version 21. Path analysis was done using Stata 14.2 to check causal effect of IBM constructs on TSE. Descriptive statistical analysis like frequency and percentage for the categorical variables and mean, standard deviation and percentage for continues variables were done. The association between the intention and each constructs of IBM will be checked using Pearson’s correlation coefficient(r). T-test and chi square were also used as needed to check the association between each construct of IBM and intention to TSE and between knowledge, environmental constrain and TSE.

Linear and logistic regressions was performed to determine whether the constructs of IBM can predict BI and TSE respectively. The result of the OR was used for interpretation of strength of prediction of the independent variables to the outcome. For all statistical significance tests, the cut- off value set was *p* < 0.05 with Confidence interval of 95%.

## Result

### Reliability of the instrument

Reliability analysis is conducted to check internal consistency of the measurement of this study using Cronbach’s alpha (α). Based on this analysis, reliability score of SE (α = 0.92) is highest, followed by DN (α = 0.89), IA (α = 0.87) after dropping one item, IN (α = 0.86), BI (α = 0.85), EA (α = 0.72) after dropping two items and PC (α = 0.71) the lowest (Table 1).

**Table 1:** Internal consistency of constructs of integrated behavioral model

Constructs	α
Experiential Attitude	0.72
Instrumental Attitude	0.87
Injunctive Norm	0.86
Descriptive Norm	0.89
Perceived Control	0.71
Self-Efficacy	0.92
Behavioral Intention	0.85

### Participants sociodemographic characteristics

Making response rate 93.6%, a total of 828 students participated in this study. The mean age was 22.5 (SD ± 2.34). Seven hundred ninety-nine (96.5%) of the participants were single. Large (85.5%) number of the respondents were orthodox in their religion. Regarding the ethnicity, highest proportion (67.5%) of ethnic group was Amhara followed by Oromo (12.4%). Around half (49%) of them were from medical and health science college and one third (32.2%) of participant’s year of study was year four. Concerning the educational status of their family, majority (39.7%) of their mother were illiterate while one third (34.6%) of their father able to read and write. Four hundred sixty-nine (56.6%) of participant’s family live in the rural area (Table 2).

**Table 2:** Socio demographic characteristics of Bahir Dar University male students

Variable	Frequency	Percent
Age Mean 22.5 ± 2.34 SD		
Marital Status		
Single	799	96.5
Ever Married	29	3.5
Religion		
Orthodox	708	85.5
Protestant	64	7.7
Muslim	49	5.9
Other	7	9
Ethnicity		
Amhara	559	67.5
Oromo	103	12.4
Tigre	80	9.7
South nations	46	5.6
Others	40	4.8
Academic Unit		
Medical and health science	406	49
Low	145	17.5
Food and Chemical	277	33.5
Year of study		
One	80	9.7
Two	161	19.5
Three	180	21.7
Four	267	32.2
Five	140	16.9
Educational status of Mother		
Illiterate	329	39.7
Able to read and write	254	30.7
Elementary School	79	9.6
Secondary School	92	11.1
College and Above	74	8.9
Educational status of father		
Illiterate	192	23.2
Able to read and write	287	34.6
Elementary School	95	11.5
Secondary School	104	12.6
College and Above	150	18.1
Family Place of Residence		
Urban	359	43.4
Rural	469	56.6

September, 2016. *N* = 828

### Testicular cancer and testicular self-examination knowledge and practice

Two third (66.8%) and three in four (41.5%) of participants said they ever heard of TC and TSE respectively. The magnitude of TSE practice in last 12 month was only 98 (11.8%). Of those who practiced TSE, none of them have performed it regularly. Few (17.1%) of participant reported they have know-how of performing TSE. One third (33.6%) of participants were knowledgeable about TSE. While more than half (55%) of them were knowledgeable about testicular cancer. More than half (54.3%) of them said there is no environmental constraint for TSE (Table 3).

**Table 3:** Knowledge and practice of Testicular self-examination among Bahir Dar University male students

Variables	Frequency	Percent
Heard of testicular cancer		
No	275	33.2
Yes	553	66.8
Heard of testicular self-examination		
No	198	23.9
Yes	630	76.1
Know-how of performing testicular self-examination		
No	686	82.9
Yes	142	17.1
Knowledge about TC		
Not Knowledgeable	373	45
Knowledgeable	455	55
Knowledge about TSE		
Not Knowledgeable	550	66.4
Knowledgeable	278	33.6
Self-Examined Testicle for TC in last 12 month		
No	730	88.2
Yes	98	11.8
Monthly performed TSE in last 12 month		
No	98	100
Yes	0	0
Environmental constraint		
No	450	54.3
Yes	378	45.7

September, 2016. *N* = 828

### Association between intention to TSE and constructs of IBM

Linear regression was performed to predict the Intention to TSE using constructs of IBM. In this model, 44.6% (Adjusted R^2^ = 44.6) of the variance explained. Experiential attitude (β = 0.33, *P* < 0.001), instrumental attitude (β = 0.12, P < 0.001) and self-efficacy (β = 0.36, P < 0.001) have positive significant association with BI. This implies that for a unit positive change in experiential attitude, instrumental attitude and self-efficacy, the BI will increase by 0.33, 0.12 and 0.36 respectively. While PC (β = −0.08, *P* < 0.05) negatively associated with BI. It appeared that a unit decrease of perceived control, the intention to TSE increase by 0.08 (Table 4).

**Table 4:** Association between behavioral intention and constructs of IBM among Bahir Dar University students September, 2016

IBM constructs	Mean	SD	Beta	*P* value	95% CI	
					Lower	Upper
Experiential Attitude	9.8	2.4	0.33	0.001*	***0.39***	***0.58***
Instrumental Attitude	21.2	4.8	0.12	0.001*	***0.04***	***0.14***
Injunctive Norm	13.6	4.0	0.02	0.66	−0.05	0.07
Descriptive Norm	12.6	4.1	0.01	0.71	−0.04	0.06
Perceived Control	9.05	2.4	−0.08	0.004*	***−0.02***	***−0.04***
Self-efficacy	31.03	8.05	0.36	0.001*	***0.13***	***0.19***

* and italics indicates significance at *p*-value < 0.05, Adjusted R^2^ = 44.6

### Factors affecting testicular self-examination of university students

Multiple logistic regression was performed to see the effect of independent variable on TSE. In this model, students’ academic unit and their father educational status from sociodemographic variables, the Know-how, knowledge about TSE, BI, injunctive norm, personal control and self-efficacy from IBM constructs showed statistical significant association with TSE and 32.6% (Nagelkerke pseudo R^2^) of the variance is explained collectively.

Students who are from Food and Chemical Engineering academic unit are 40% less likely to perform TSE as compared with those from medical and health science academic unit **[AOR = 0.40; CI: 0.18–0.85]**. Those whom their father able to read and write and completed elementary school were 2.25 and 3 times more likely to perform TSE as compared with those whom their father is illiterate **[AOR = 2.25; CI: 1.15–4.44]** and **[AOR = 3.00; CI: 1.36–6.64]** respectively.

Student who have Know-how of performing TSE are 3.35 times more likely to perform it as compared with those who do not have Know-how of performing it **[AOR = 3.35; CI: 1.94–5.80**]. participant students who are knowledgeable about TSE are 3.93 times more likely to perform TSE as compared with those who are not knowledgeable **[AOR = 3.93; CI: 2.30–6.72]**. A unit increase in intention to TSE lead an increase TSE practice by 20% [**AOR = 1.20; CI:1.10–1.31**]. As injunctive norm decreases by one unit, the odds of TSE practice increase by 11% [**AOR = 0.89; CI:0.82–0.96**]. One unit decrease in personal control lead to 10% increase in TSE [**AOR = 0.90; CI: 0.82–0.92**]. One unit increase of self-efficacy increases the TSE practice by 6% [**AOR = 1.06; CI: 1.02–1.11**] (Table 5).

**Table 5:** Factors affecting testicular self-examination of Bahir Dar University male students September, 2016

Variable	TSE		Adjusted Odd Ratio	95% CI	
	Beta	Wald		Lower	Upper
Academic Unit					
Medical & health science		6.25	1	1	1
Low	0.43	0.02	1.04	0.52	2.09
Food and Chemical engineering	−0.91	5.60	0.40*	***0.18***	***0.85***
Educational status of Father					
Illiterate		12.43	1	1	1
Able to read and write	0.81	5.56	2. 25*	***1.15***	***4.44***
Elementary School	1.10	7.41	3.00*	***1. 36***	***6.64***
Secondary School	0.14	0.07	1.15	0.42	3.16
College and Above	0.07	0.03	1.08	0.45	2.58
Know-how to perform TSE					
No			1	1	1
Yes	1.21	18.74	3.35*	***1.94***	***5.80***
Knowledge					
Not knowledgeable			1	1	
Knowledgeable	1.37	25.32	3.93*	***2.30***	***6.72***
Environmental constraint					
No		1.08	1	1	1
Yes	0.43	2.16	1.54	0.86	2.75
Behavioral Intention	0.18	13.80	1.20*	***1.09***	***1.32***
Experiential Attitude	−0.08	1.37	0. 24	0.81	1.05
Instrumental Attitude	−0.04	0.88	0.96	0. 89	1.04
Injunctive Norm	−0.11	7.22	0.89*	***0.82***	***0.96***
Descriptive Norm	0.02	0.05	1.02	0.95	1.10
Perceived Control	−0.06	1.53	0.90*	***0.82***	***0.92***
Self-Efficacy	0.06	8.91	1.06*	***1.02***	***1.11***

* and italics indicates significance at *P*-value <0.05, Adjusted R^2^ = 32.6 embedded

### Path analysis of IBM

**Fig. 1:**
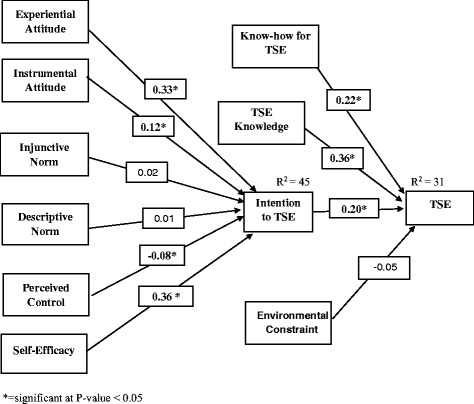
Path model of testicular self-examination among Bahir Dar university students

Intention to TSE (endogenous variable) was assessed using experiential attitude, instrumental attitude, injunctive norm, descriptive norm, personal control and self-efficacy as the exogenous variables. Four constructs; experiential attitude, instrumental attitude, personal control, and self-efficacy proved statistically significant (*p* < 0.05) with path coefficients of 0.33, 0.12, −0.08 and 0.36 respectively. The IBM accounted for 45% of the variance (R^2^) in the intention to perform TSE. In this analysis, TSE examination was functioned as the endogenous variable and BI, EC, Knowledge and Know-how to performing TSE were used as exogenous variables to predict TSE. BI, knowledge and Know-how to perform TSE were statistically significant (*P* < 0.05) with path coefficients of 0.2, 0.36 and 0.22 respectively. The model accounted for 31% of the variance (R^2^) in TSE (Fig. 1).

Generally, the Bentler’s Comparative Fit (CFI) Index (0.95), Joreskog-Sorbom’s Goodness of Fit (GFI) Index (.93) and Root Mean-Square Error of Approximation (RMSEA) of 0.062 with its 90% confidence interval of 0.043–0.084 proved the model used to predict the behavioral intention and binge drinking based on IBM constructs showed an acceptable model fit indices.

## Discussion

In this study, we were able to identify a low TSE practice among University students, with 11.8% of them practicing in the last 12 months. This finding is comparable with studies conducted in different places. In study conducted in turkey among university students showed 17.7% of student who ever performed TSE [11]. In another study conducted among university students from low income (Bangladesh and Madagascar), middle income (South Africa and Turkey) and emerging economy countries, 13.6% of the students ever practiced TSE in last 12 months [9].

In contrast, the current finding is very low as compared with some studies of developed countries conducted before a decade; though the findings are not remarkably high. In study conducted in Australia, 44.6% of the university students performs TSE in a year. In two studies conducted in UK among patients attending General Practice and University students, 49 and 59% of the participants practiced TSE respectively [10, 16, 17]. The difference might be availability of TC related health campaign which improved the level of their awareness, knowledge, attitude and intention; as these variables have direct relationship with TSE in these studies. Hence, we suggest the implementation of health campaign on TC and TSE in current study area.

Additionally, none of participants of this study performed TSE regularly. In study conducted in Nigeria there is also no individual who performed TSE monthly. The finding of study in UK among undergraduate and postgraduate student also revealed only one individual performed it regularly [18, 19]^.^ Though their finding were still not high enough., there is big difference as compared with other studies; The finding of study conducted in Australia, 17.8% of the university students performs TSE regularly. In those of two studies conducted in United Kingdom (UK) among patients attending General Practice and University students, 22% and 5% of the participants practiced TSE respectively [10, 16, 17]. It might be implementation of TC related public health campaign in these study areas for this difference.

The proportion of student who heard about TC and TSE is 59.8% and 34.9% respectively. This is higher as compared with two study conducted in Nigeria. In study conducted among college students, only 10.4 and 1% of the students aware of TC and TSE. In that of study among high school boys, only 1% of them heard about TSE [19, 20].This difference is due to number of study participants who are health science students; which is around half of sample in current study. However, it is lower as compared with study conducted almost two decades ago in UK and Australia. The proportion of participants who heard of was 90.6% in that of UK study. In the study among Australia University. 58.4% of the participant heard of TSE [17, 18]. This indicates big difference in availability of health information between developed and developing countries even after long period of time.

Of the six constructs of Integrated Behavioral Model, experiential attitude, instrumental attitude, personal control and self-efficacy have statistically significant association with intention to TSE with beta coefficient 0.33, 0.12, −0.08 and 0.36 respectively. In study which used IMB to investigate predictor of binge drinking behavior of university students, experiential attitude, injunctive norm and self-efficacy were significantly associated with BI [21]. SE and PC were significant predictor of intention in study which investigated TSE using TPB as a conceptual framework [10]. In addition to attitude and subjective norm, PC and SE were also found predictor of breast self-examination and TSE intention in study which investigated the value of TPB [14]. Self-efficacy has been found significant determinant of BI in studies conducted to assess the health protective behavior [15, 22, 23]. However descriptive and injunctive norm were not predictive of intention to TSE in current study. This indicates referent others approval or disproval have no effect on intention to TSE among these students. The possible reason for this could be due to variation across the behavior, population and situation under which behavior is occurring according to IBM perspective [13].

The path analysis also revealed the same finding in which, experiential attitude, instrumental attitude, personal control and self-efficacy have statistically significant association with BI showing path coefficient of 0.33, 0.12, −0.08 and 0.36 respectively and path from BI to TSE showed path coefficient of 0.36. In study conducted to test the TPB and the health belief model (HBM) in predicting testicular self-examination (TSE) behavior, self-efficacy and perceived behavioral control have statistically significant association with intention showing path coefficient 0f 0.83 and - 0.28 respectively as the path from intention to behavior was β = 0.41 [14]. In study conducted to predict binge drinking, experiential attitude, injunctive norm and self-efficacy were statistically significant to BI with path coefficients of 0.34, 0.23, and −0.27, respectively and the path from intention to behavior was 0.03 [21].

BI, knowledge about TSE and Know-how to perform TSE were significantly associated with TSE. The relationship between these variables and TSE is also proved in path analysis. The significance of the BI on behavior is being evidenced by finding of different researchers [10, 24]. Though Environmental Constraint have no significant association with behavior, significance of Knowledge and Know-how to perform behavior has proved the assumption of IBM about the importance of these factors for behavior to occur in addition to BI [13].

IBM explained 44.6% and 32.6% of variance for BI and TSE in regression respectively. The finding of path analysis also showed that 45% of BI and 31% of TSE in current study. It is comparable similar with study done on binge drinking in which 44% of BI and 26% of behavior explained by model [21]. The finding of meta-analysis which revealed 39 and 27% of the variance in BI and behavior respectively is also comparable with current study [25]. A major limitation of this study was the utilization of cross-sectional design; which limit not only reporting causal inferences but also measuring temporal stability (test-retest reliability) of the construct. Since the intention, behavior and constructs IBM do not change concurrently, prospective study design is recommended when IBM is used as conceptual frame work to measure the intention of a behavior and the behavioral performance at two separate points in time; but, due to a lack of resources and time, the current study did not employ that type of research design Future study should examine the causal relationship of the variables using analytical study design.

## Conclusion

The finding of this study revealed that practice of TSE among university students is low. None of the student regularly practice TSE. Knowledge about TC and TSE is low among the students. BI is found as the result of EA, IA, SE and PC. BI, being knowledgeable about and having Know-how of performing TSE have significant effect on TSE. Hence, making information about TC and TSE available for students and implementing behavior change Communication strategy that focus on identified behavioral factors should be designed and implemented to improve university students regular TSE practice.

## Additional file

Additional file 1: Survey instrument used to assess testicular self examination among Bahir Dar University students. (DOCX 34 kb)

## Abbreviations

BI: Behavioral Intention
DN: Descriptive Norm
EA: Experiential Attitude
EC: Environmental Constraint
IA: Instrumental Attitude
IN: Injunctive Norm
OR: Odd Ratio
PC: Perceived Control
SE: Self Efficacy
SPSS: Statistical Package for Social Science
TC: Testicular cancer
TPB: Theory of planned behavior
TSE: Testicular Self Examination
