# Supplementary material for: Testicular self examination among Bahir Dar University students: application of integrated behavioral model
Source: BMC Cancer. 2018 Jan 4;18:21. doi: 10.1186/s12885-017-3935-8 (PMC5755162; doi:10.1186/s12885-017-3935-8)
Supplement: Additional file 1: — Survey instrument used to assess testicular self examination among Bahir Dar University students. (DOCX 34 kb) [file 12885_2017_3935_MOESM1_ESM.docx]

Interview No ------------------- Date of interview -------------------------

Respondent’s college/faculty/school ----------------- Respondent’s Department -------------

**Part I. Socio –Demographic characteristics**

| Code | Questions | Possible responses |
| --- | --- | --- |
| 101 | How old are you? | 1. ------ years |
| 102 | What is your current Marital status? | 1. single  2. Married  3. Divorced  4. Widowed  99. Others, Specify ---------- |
| 103 | What is your religion? | 1. Orthodox  2. Protestant  3. Muslim  4. Catholic  99.others,specify ________ |
| 104 | To which ethnicity do you belong? | 1. Amhara  2. Awi  3. Tigrie  4. Oromo  99.Others,specify________ |
| 105 | What is your year of study | ----------------------- |
| 106 | Educational status of your father | 1. illiterate  2. Able to read and write  3. Elementary school  4. secondary school  5. grade 12 complete  6. College and above |
| 107 | Educational status of your mother | 1. illiterate  2. Able to read and write  3. Elementary school  4. secondary school  5. grade 12 complete  6. College and above |
| 108 | Family monthly income | _________(Birr) |
| 109 | Family place of residence? | 1. Urban 2. Rural |

**Part** II: **Knowledge and practice related question**

| **s. no** | Question | Response | Skip |
| --- | --- | --- | --- |
| 201 | Do you know how to perform testicular self-examination | 1. Yes 2. No |  |
| 202 | Have ever examine your testicles for testicular cancer? | 1. Yes 2. No | To 203 if 2 |
| 203 | If yes to 201, how many times during the past year did you examine your testicles for testicular cancer? | ------------------------ |  |
| 204 | Have you ever heard of testicular cancer? | 1. Yes 2. No | To 205 if 2 |
| 205 | If yes to 203, from where did get information? (multiple answer is possible) | ------------------------ |  |
| 206 | Have you ever heard of testicular self-examination? | 1. Yes 2. No | To 207 if 2 |
| 207 | If yes to 205, from where did get information? (multiple answer is possible) | ---------------------- |  |
| 208 | Testicular Cancer is most common in men aged 15-45 years. | 1. True 2. False |  |
| 209 | Any pain in the testicle is the symptom of Testicular Cancer. | 1. True 2. False |  |
| 210 | A lump in the testicle is the symptom of Testicular Cancer. | 1. True 2. False |  |
| 211 | Testicular cancer is almost always curable if found early. | 1. True 2. False |  |
| 212 | Treatment of testicular cancer usually starts with surgery to remove the testicle with cancer. | 1. True 2. False |  |
| 213 | The people who has Testicular Cancer can’t have any children | 1. True 2. False |  |
| 214 | Testicular examination is only done by skilled professionals. | 1. Yes 2. No |  |
| 215 | Testicular Self Examination helps detecting the masses in the testis. | 1. True 2. False |  |
| 216 | Self-examination once a month help to detect TC early. | 1. True 2. False |  |
| 217 | Convenient time to examine testicle is while taking shower with warmth water. | 1. Yes 2. No |  |
| 218 | Testicular Self Examination is painful | 1. True 2. False |  |

**Part III: Behavior related statements**

| **s.no** | **Statements** | **Strongly disagree (1)** | **Disagree (2)** | **Neutral (3)** | | **Agree (4)** | **Strongly agree (5)** |
| --- | --- | --- | --- | --- | --- | --- | --- |
| 301 | I intend to perform testicular self-examination once a month. |  |  |  | |  |  |
| 302 | I will perform testicular self-examination in the next month. |  |  |  | |  |  |
| 303 | I have decided to perform testicular self-examination in the next month. |  |  |  | |  |  |
| 304 | I want to perform testicular self-examination regularly each month. |  |  |  | |  |  |
| 405 | If I perform testicular self-examination monthly, I will feel happy. |  |  |  | |  |  |
| 306 | If I perform testicular self-examination monthly, I will never afraid I have developed testicular cancer. |  |  |  | |  |  |
| 307 | Performing testicular self-examination will embarrasses me |  |  |  | |  |  |
| 308 | Performing testicular self-examination every month is not interesting for me. |  |  |  | |  |  |
| 309 | Performing testicular self-examination makes me feel healthy |  |  |  | |  |  |
| 310 | If I do monthly self-examinations, I may find a lump in my testicle |  |  |  | |  |  |
| 311 | Performing testicular self-examination helps me to search for further health check-ups. |  |  |  | |  |  |
| 312 | My performing of testicular self-examination helps me for detection of testicular cancer in early stage. |  |  |  | |  |  |
| 313 | Detecting testicular cancer by performing testicular self-examination make the cure faster. |  |  |  | |  |  |
| 314 | Early detection of testicular cancer by performing testicular self-examination increase the chance of survival. |  |  |  | |  |  |
| 315 | Treatment and early detection of testicular cancer by performing testicular self-examination minimize the chance of developing prostate cancer. |  |  |  | |  |  |
| 316 | If I perform testicular self-examination monthly, I am doing something important for my health. |  |  |  | |  |  |
| 317 | Most people who are important to me think that I should perform testicular self-examination once a month. |  |  |  | |  |  |
| 318 | Most people who are important to me would want of me performing testicular self-examination once a month. |  |  |  | |  |  |
| 319 | The people in my life whose opinion I value would approve me to perform testicular self-examination once a month. |  |  |  | |  |  |
| 320 | My family thinks that I should do Testicular self-examination monthly. |  |  |  | |  |  |
| 321 | My dorm-mates thinks that I should do Testicular self-examination monthly. |  |  |  | |  |  |
| **S.no** | **Statements** | **Strongly disagree (1)** | **Disagree (2)** | **Neutral (3)** | | **Agree (4)** | **Strongly agree (5)** |
| 322 | My best friends thinks that I should do Testicular self-examination monthly. |  |  |  | |  |  |
| 323 | My girlfriend thinks that I should do Testicular self-examination monthly. |  |  |  | |  |  |
| 324 | My classmates thinks that I should do Testicular self-examination monthly. |  |  |  | |  |  |
| 325 | Most people who are important to me perform testicular self-examination once a month |  |  |  | |  |  |
| 326 | People in my life whose opinion I value perform testicular self-examination once a month. |  |  |  | |  |  |
| 327 | Most of university students perform testicular self-examination once a month. |  |  |  | |  |  |
| 328 | My father perform Testicular self-examination monthly. |  |  |  | |  |  |
| 329 | My classmates do Testicular self-examination once a month. |  |  |  | |  |  |
| 330 | My dorm- mates do Testicular self-examination monthly. |  |  |  | |  |  |
| 331 | My brother perform Testicular self-examination monthly. |  |  |  | |  |  |
| 332 | My best friends do Testicular self-examination monthly. |  |  |  | |  |  |
| 333 | I expect that taking modular course will place high demands on my time. |  |  |  | |  |  |
| 334 | Reminder should present to perform testicular self-examination regularly. |  |  |  | |  |  |
| 335 | I couldn’t get warmth shower in the campus if I wanted to. |  |  |  | |  |  |
| 336 | I belief special skill is required to perform testicular self-examination. |  |  |  | |  |  |
| 337 | Machines are required to perform testicular self-examination |  |  |  | |  |  |
| 338 | I am confident that I can perform testicular self-examination once a month. |  |  |  | |  |  |
| 339 | I believe I have the ability to perform testicular self-examination once a month. |  |  |  | |  |  |
| 340 | I can perform testicular self-examination even if I feel uncomfortable about it. |  |  |  | |  |  |
| 341 | I can perform testicular self-examination even if I have to seek out information about the technique. |  |  |  | |  |  |
| 342 | I feel capable of performing testicular self-examination once a month. |  |  |  | |  |  |
| 343 | I am confident that I can perform testicular self-examination once a month even if warmth shower not available in the campus. |  |  |  | |  |  |
| 344 | I am sure I can perform testicular self-examination once a month by adjusting my demanding time. |  |  |  | |  |  |
| 345 | I am sure I can perform testicular self-examination regularly even if nothing is found when I perform last time. |  |  |  | |  |  |
| 346 | I am sure I can perform testicular self-examination regularly When there is no remainder |  |  |  | |  |  |
| 347 | I am confident that I can perform testicular self-examination once a month even if I am afraid to find something in testicle |  |  |  | |  |  |
| 348 | How certain you could be to perform testicular self-examination monthly?  1. Extremely certain I could not  2. Certain I could not  3. Neutral  4. Certain I could  5. Extremely certain I could | | | |  |  |  |
| 349 | There is no access to warmth shower for the students in the campus.   1. Yes 2. No | | | |  |  |  |
| 350 | There is no reminder about the testicular self- examination for the students.   1. Yes 2. No | | | |  |  |  |
| 351 | The shower in the campus is convenient place to perform testicular self-examination   1. Yes 2. No | | | |  |  |  |
| 352 | There is a program which is working to make students aware of testicular cancer and motivating them to start performing testicular self-examination in BDU.   1. Yes 2. No | | | |  |  |  |

**Performing testicular self - examination is:-**

Harmful : 1 : 2 : 3 : 4 5: 6: 7 : Beneficial

Pleasant : 1 : 2 : 3 : 4 5: 6: 7 : Unpleasant

Good : 1 : 2 : 3 : 4 5: 6: 7 : Bad

Valueless : 1 : 2 : 3 : 4 5: 6: 7 : Valuable

Enjoyable : 1 : 2 : 3 : 4 5: 6: 7 : Unenjoyable

Uncomfortable : 1 : 2 : 3 : 4 5: 6: 7 : comfortable

Fast : 1 : 2 : 3 : 4 5: 6: 7 : Slow

Stressful : 1 : 2 : 3 : 4 5: 6: 7 : Relaxing

Shy : 1 : 2 : 3 : 4 5: 6: 7 : Confident

Foolish : 1 : 2 : 3 : 4 5: 6: 7 : Wise

Risky : 1 : 2 : 3 : 4 5: 6: 7 : Safe

Boring : 1 : 2 : 3 : 4 5: 6: 7 : Fun

Easy : 1 : 2 : 3 : 4 5: 6: 7 : Difficult

Cheap : 1 : 2 : 3 : 4 5: 6: 7 : Expensive

Controllable : 1 : 2 : 3 : 4 5: 6: 7 : Uncontrollable
